# Supplementary material for: ST3 beta-galactoside alpha-2,3-sialyltransferase 1 (ST3Gal1) synthesis of Siglec ligands mediates anti-tumour immunity in prostate cancer
Source: Commun Biol. 2024 Mar 6;7:276. doi: 10.1038/s42003-024-05924-0 (PMC10918101; doi:10.1038/s42003-024-05924-0)
Supplement: Supplementary file 2 — Supplementary Information [file 42003_2024_5924_MOESM2_ESM.pdf]

Supplementary Figure 1.

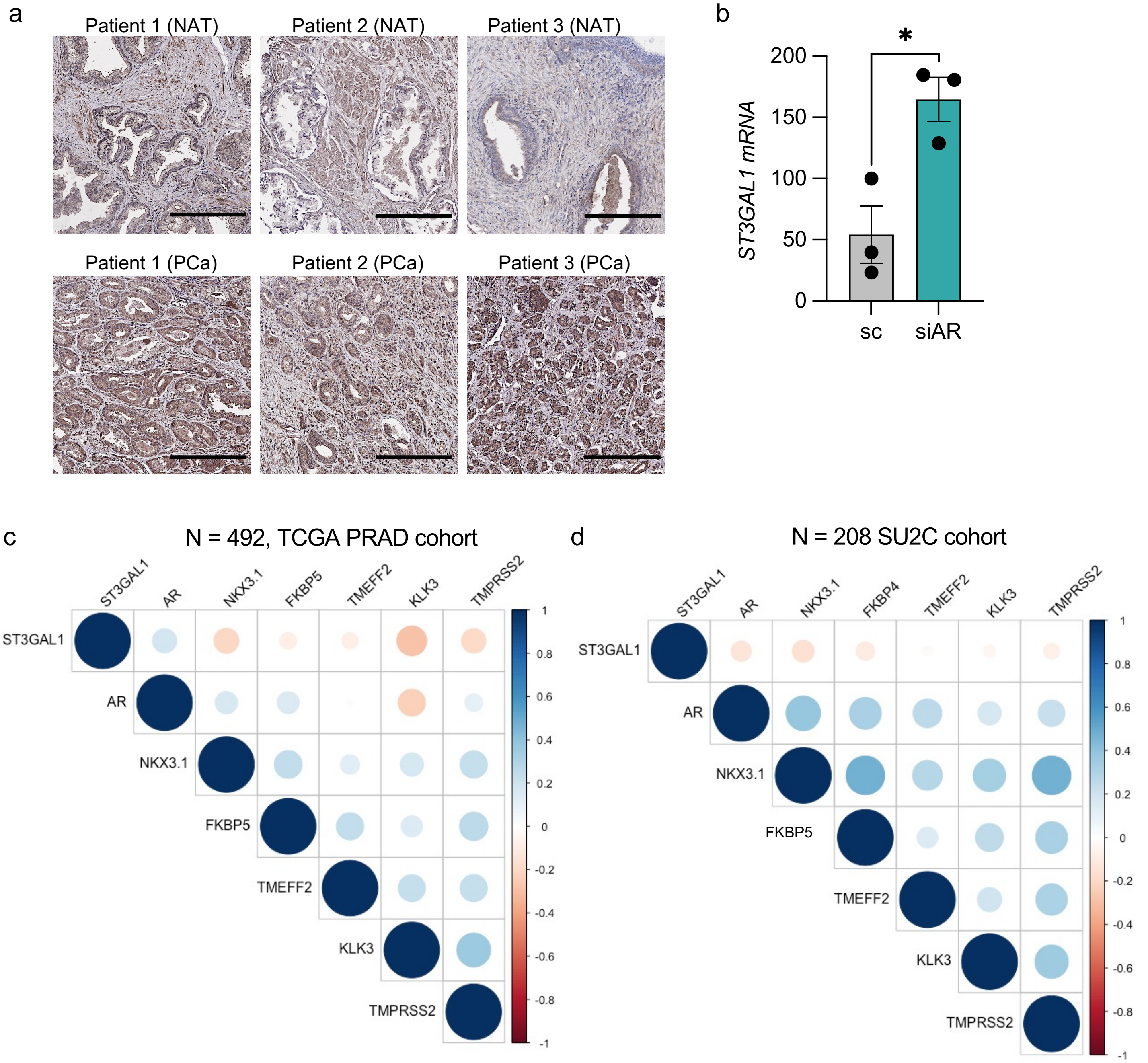

(a) ST3Gal1 is upregulated in prostate cancer and negatively correlates with androgen receptor signalling. Additional representative images of ST3Gal1 protein expression shown using immunohistochemistry in normal adjacent tissue (NAT) or prostate cancer (PCa). Scale bar = 300 μM. (b) Quantification of *ST3GAL1* mRNA by RT-qPCR in LNCaP cells following siRNA knockdown of full-length AR. (c) Correlation matrix correlogram showing *ST3GAL1* gene in prostate cancer patients (N=492). Pearson's correlation coefficient is shown from −1 (red) to 1 (blue). Only correlations with statistical significance of $p < 0.05$ are shown. The size of the circle is proportional to the correlation coefficients. (d) Correlation matrix correlogram showing *ST3GAL1* gene in CRPC patients (N=208). Pearson's correlation coefficient is shown from −1 (red) to 1 (blue). Only correlations with statistical significance of $p < 0.05$ are shown. The size of the circle is proportional to the correlation coefficients.

Supplementary Figure 2.

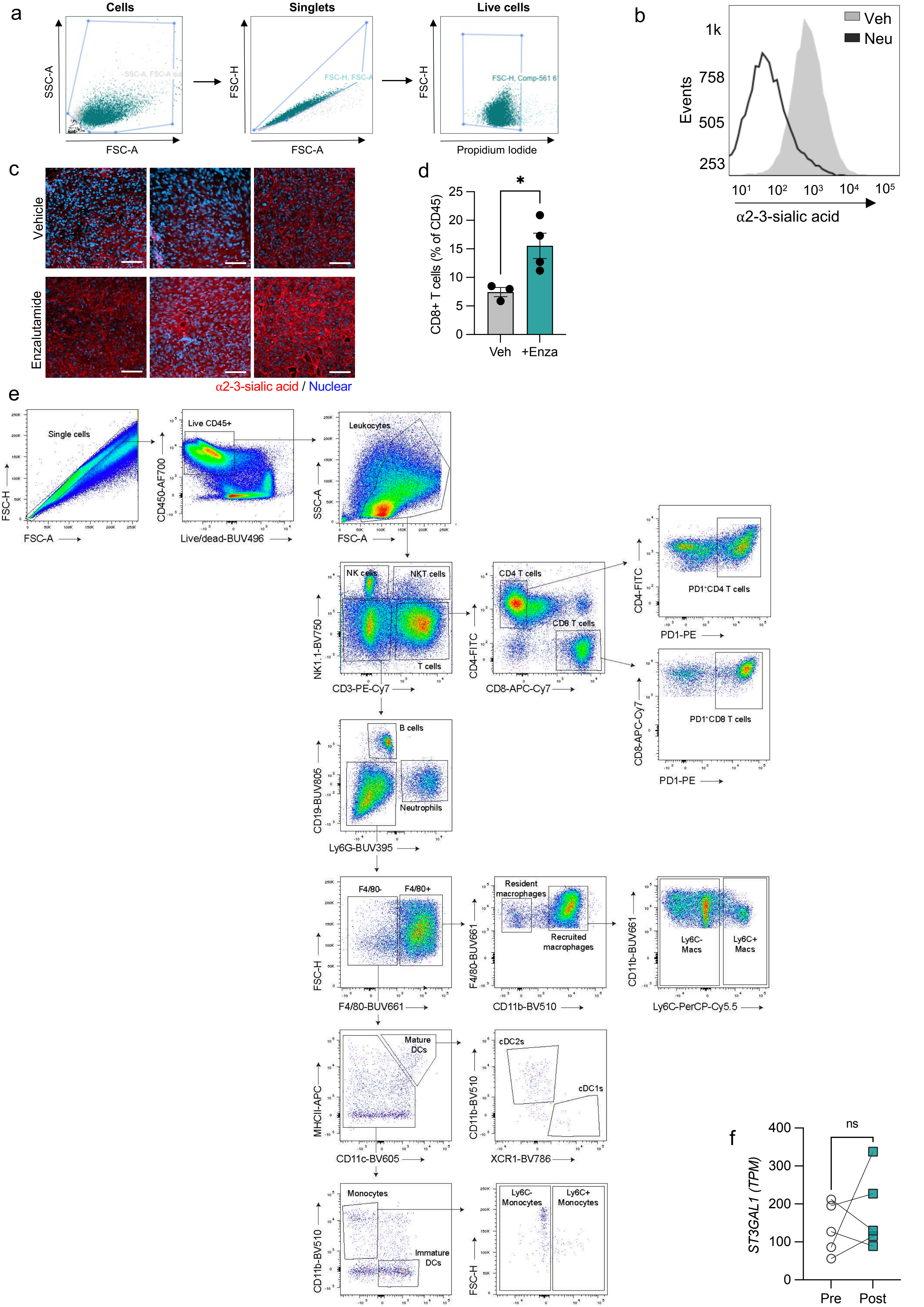

(a) Gating strategies for flow cytometry and representative images of MAL-II staining of mouse tumours. Schematic showing the flow cytometry gating strategy used for all MAL-II and Siglec-FC flow cytometry experiments. (b) Representative histogram of MAL-II lectin detection of α2'3-sialylation in LNCaP cells following following neuraminidase treatment. (c) Additional representative images of MAL-II lectin immunofluorescence detection of α2-3- linked sialic acid (red) expression in FFPE subcutaneous TRAMP-C2 tumours treated with vehicle or enzalutamide. Scale bar = 100 μM. (d) Number of $CD8^{+}$ T cells in TRAMP-C2 allografts following 20 mg/kg enzalutamide treatment for 7 days. Shown as a percentage of the total $CD45^{+}$ population. (e) Example of the flow cytometry gating strategy used in immune profiing experiments. (f) ST3Gal1 gene expression levels determined by RNA sequencing of match biopsies pre and post enzalutamide treatment (n=5).

Supplementary Figure 3.

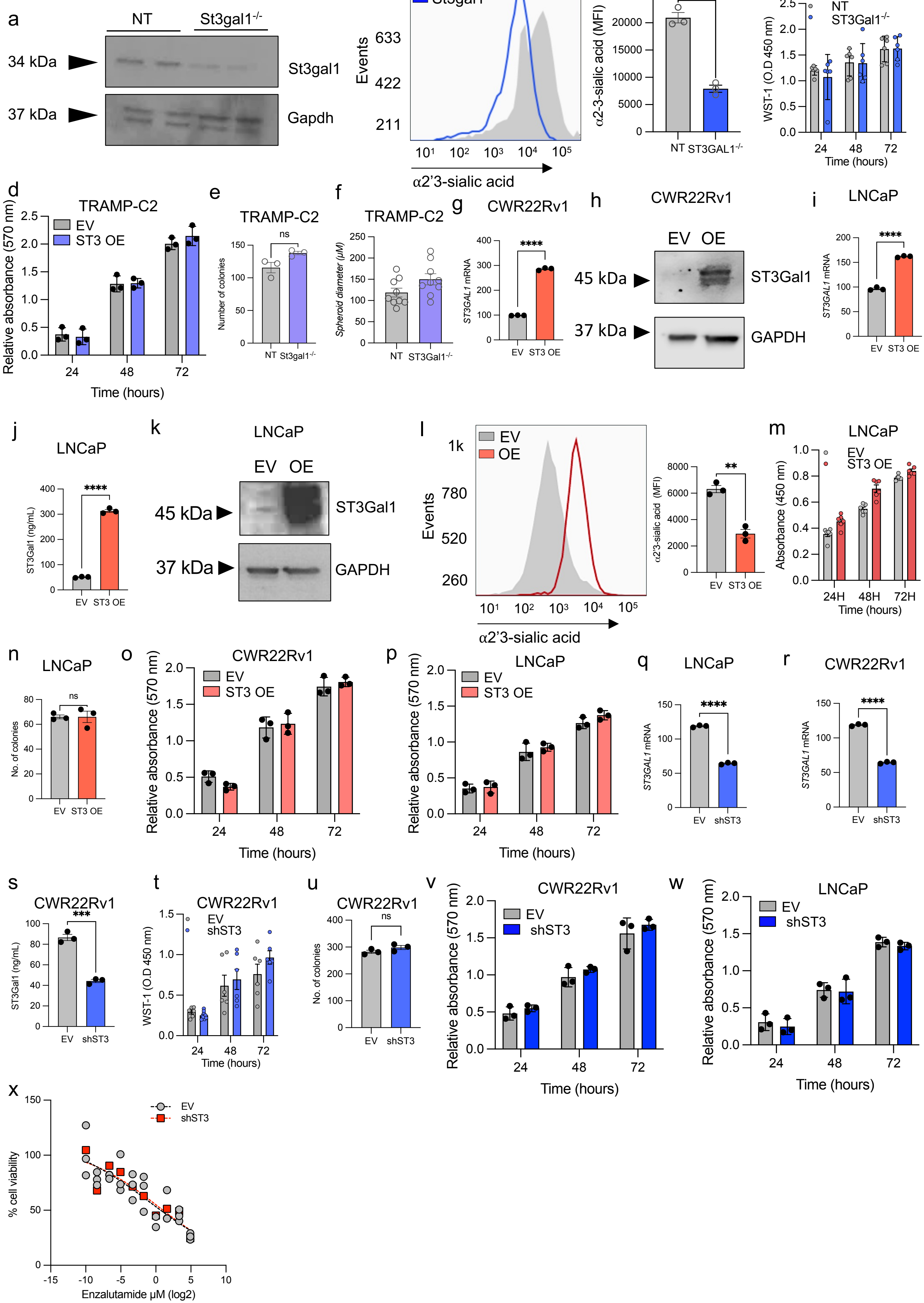

(a) ST3Gal1 does not affect the cellular behaviours of prostate cancer cells. Western blot for St3gal1 in non-targeting sgRNA TRAMP-C2 cells and St3gal1$^{-/-}$ cells. (b) MAL-II lectin detection of α2'3-sialylation in sgRNA TRAMP-C2 cells and St3gal1$^{-/-}$ cells by flow cytometry. (c) Cellular proliferation of non-targeting (NT) and St3gal1$^{-/-}$ TRAMP-C2 cells quantified by WST-1 assay. Absorbance was read at 450 nm and normalised to background absorbance. (d) Proliferation of NT and and St3gal1$^{-/-}$ cells measured using a SRB assay. Absorbance was read at 570 nm. (e) Colony forming ability of NT and St3gal1$^{-/-}$ TRAMP-C2 cells measured using a colony forming assay. Graph shows number of colonies formed. (f) Diameters of NT and St3gal1$^{-/-}$ spheroids calculated using Image J. Nine spheroids from each group were quantified. (g) RT-qPCR mRNA levels of ST3GAL1 in empty vector (EV) and ST3GAL1 overexpression (OE) lentiviral transduced CWR22Rv1 cells. (h) Western blot for ST3Gal1 in EV and OE CWR22Rv1 cells. (i) RT-qPCR mRNA levels of ST3GAL1 in EV and ST3GAL1 OE lentiviral transduced LNCaP cells. (j) Protein levels of ST3Gal1 in EV and ST3GAL1 OE lentiviral transduced LNCaP cells determined by ELISA. (k) Western blot for ST3Gal1 in EV and OE LNCaP cells. (l) MAL-II lectin detection of α2'3-sialylation in EV and OE LNCaP cells by flow cytometry. (m) Cellular proliferation of EV and OE LNCaP cells quantified by WST-1 assay. Absorbance was read at 450 nm and normalised to background absorbance. (n) Colony forming ability of EV and OE LNCaP cells measured using a colony forming assay. Graph shows number of colonies formed. (o) Proliferation of EV and ST3 OE CWR22Rv1 cells measured using a SRB assay. Absorbance was read at 570 nm. (p) Proliferation of EV and ST3 OE LNCaP cells measured using a SRB assay. Absorbance was read at 570 nm. (q) RT-qPCR mRNA levels of ST3GAL1 in EV and shST3GAL1 lentiviral transduced LNCaP cells. (r) RT-qPCR mRNA levels of ST3GAL1 in EV and shST3GAL1 lentiviral transduced CWR22Rv1 cells. (s) Protein levels of ST3Gal1 in EV and shST3GAL1 lentiviral transduced CWR22Rv1 cells determined by ELISA. (t) Cellular proliferation of EV and shST3GAL1 CWR22Rv1 cells quantified by WST-1 assay. Absorbance was read at 450 nm and normalised to background absorbance. (u) Colony forming ability of EV and shST3GAL1 CWR22Rv1 cells measured using a colony forming assay. Graph shows number of colonies formed. (v) Proliferation of EV and shST3 CWR22Rv1 cells measured using a SRB assay. Absorbance was read at 570 nm. (w) Proliferation of EV and shST3 LNCaP cells measured using a SRB assay. Absorbance was read at 570 nm. (x) Cellular viability of EV and shST3 LNCaP cells treated with a range of concentrations of enzalutamide for 72 hours. Percentage viability calculated as a percentage of vehicle control cells. Absorbance was read at 570 nm.

Supplementary Figure 4.

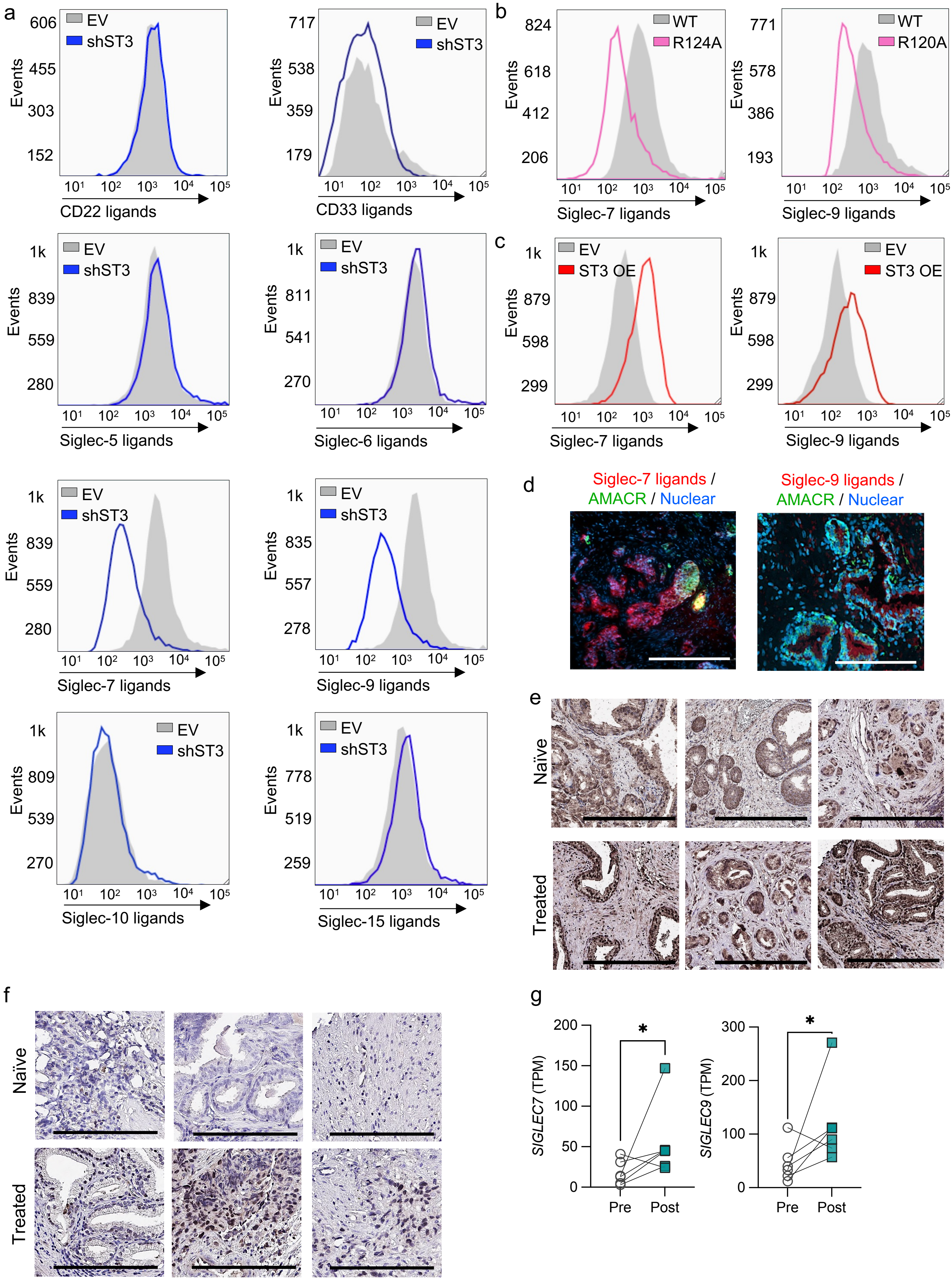

Probing Siglec ligands in prostate cancer cells by flow cytometry and in prostate cancer tissue by immunofluorescence and immunohistochemistry. (a) Representative histograms for Siglec-Fc quantification of siglec ligands on empty vector and sh*ST3GAL1* LNCaP cells as determined by flow cytometry. (b) Representative histograms for engineered Siglec-9-Fc. WT Siglec-Fc is compared with an R120A/R124A mutant with reduced Siglec binding capacity. (c) Representative flow cytometry histograms for Siglec-7hFc and Siglec-9hFc quantification of siglec ligands on empty vector and ST3Gal1 overexpressing CWR22Rv1 cells. (d) Additional representative images of Siglec-7 /9 ligands (red) colocalized with AMACR (green) in prostate cancer patient biopsies using dual immunofluorescence. Scale bar = 300 *μ*m. (e) Additional representative images of immunohistochemistry detection of Siglec-9 ligands using Siglec-Fc reagents in a tissue microarray (TMA). Patients include those who are treatment naïve (N=26) and those who have been exposed to androgen deprivation therapy (N=24). H-scores were generated to quantify staining in epithelial cells using a Leica Aperio slide scanner. Scale bar = 300 μm. (f) Additional representative images of Immunohistochemistry detection of Siglec-9 in a tissue microarray (TMA). Patients include those who are treatment naïve (N=30) and those who have been exposed to androgen deprivation therapy (N=32). The number of positive Siglec-9+ cells were quantified per tissue core. Scale bar = 200 μm. (g) *SIGLEC7* and *SIGLEC9* gene expression levels determined by RNA sequencing of match biopsies pre and post enzalutamide treatment (n=6).

Supplementary Figure 5.

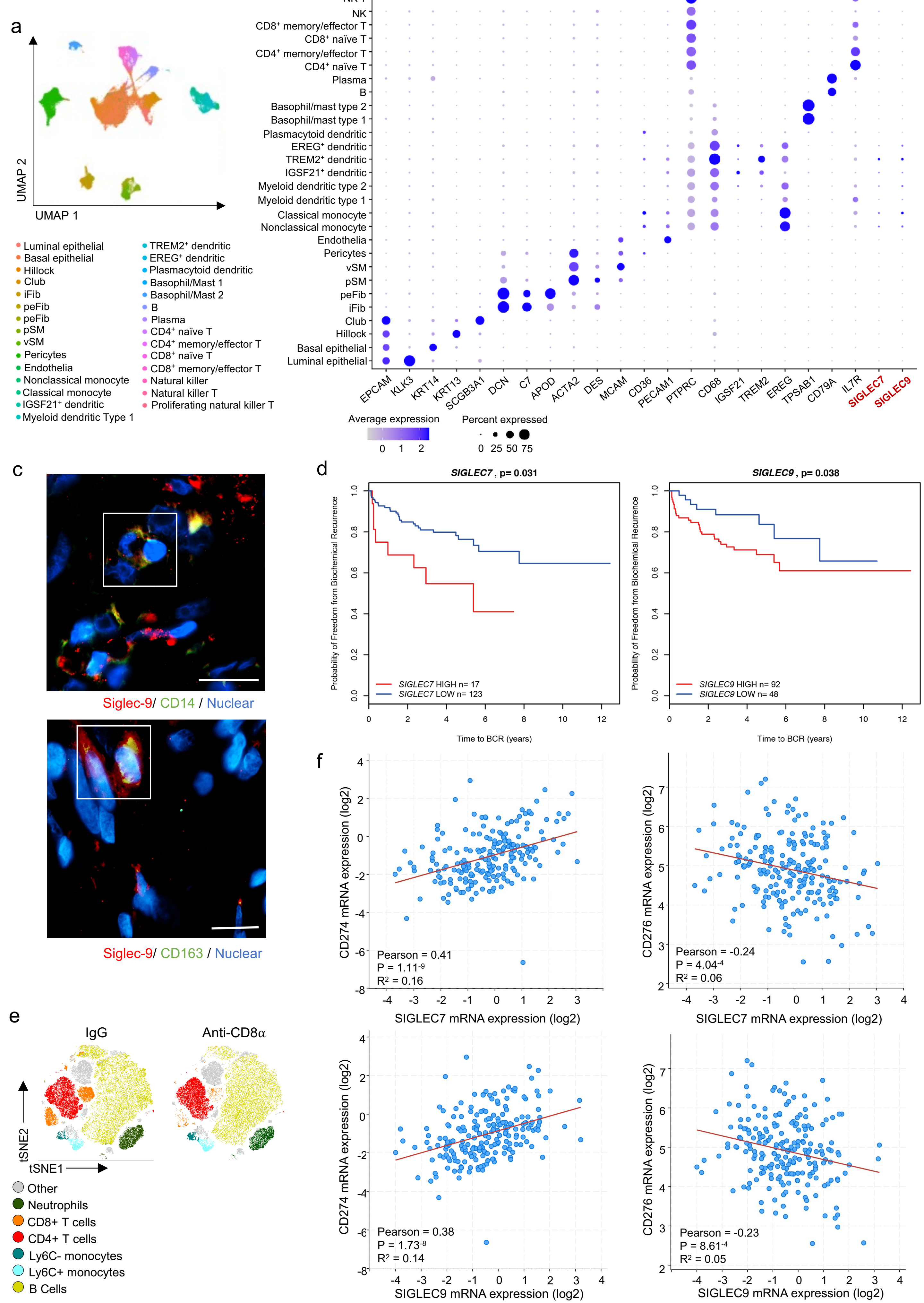

Single-cell expression of *SIGLEC7* and *SIGLEC9*, their association with survival and conventional immune checkpoints. (a) Uniform manifold approximation and projection (UMAP) showing clustering of cell types in single-cell transcriptomic analysis of human prostate. (b) Dot plot of transcriptomic expression of cell type markers and *SIGLEC7* and *SIGLEC9*. (c) Additional representative images of dual immunofluorescence staining of Siglec-9 positive (red) myeloid cells with CD14 and CD163 (green) in prostate cancer patient biopsies. Scale bars = 20 µm. (d) Kaplan–Meier plot showing time to biochemical reoccurrence for prostate cancer patients stratified based on low or high *SIGLEC7* and *SIGLEC9* gene expression. Analysis performed on the MSKCC dataset accessed through camcAPP. (e) t-distributed stochastic neighborhood embedding) tSNE maps of flow cytometry analysis of circulating immune populations in selective depletion TRAMP-C2 subcutaneous allografts treated with IgG or anti-CD8. (f) Pearson's correlation of transcript levels of *SIGLEC7* and *SIGLEC9* with CD274 and CD276 in N=208 patients with castrate resistance prostate cancer in the SU2C cohort accessed and analysed using cBioPortal.

Supplementary Figure 6.

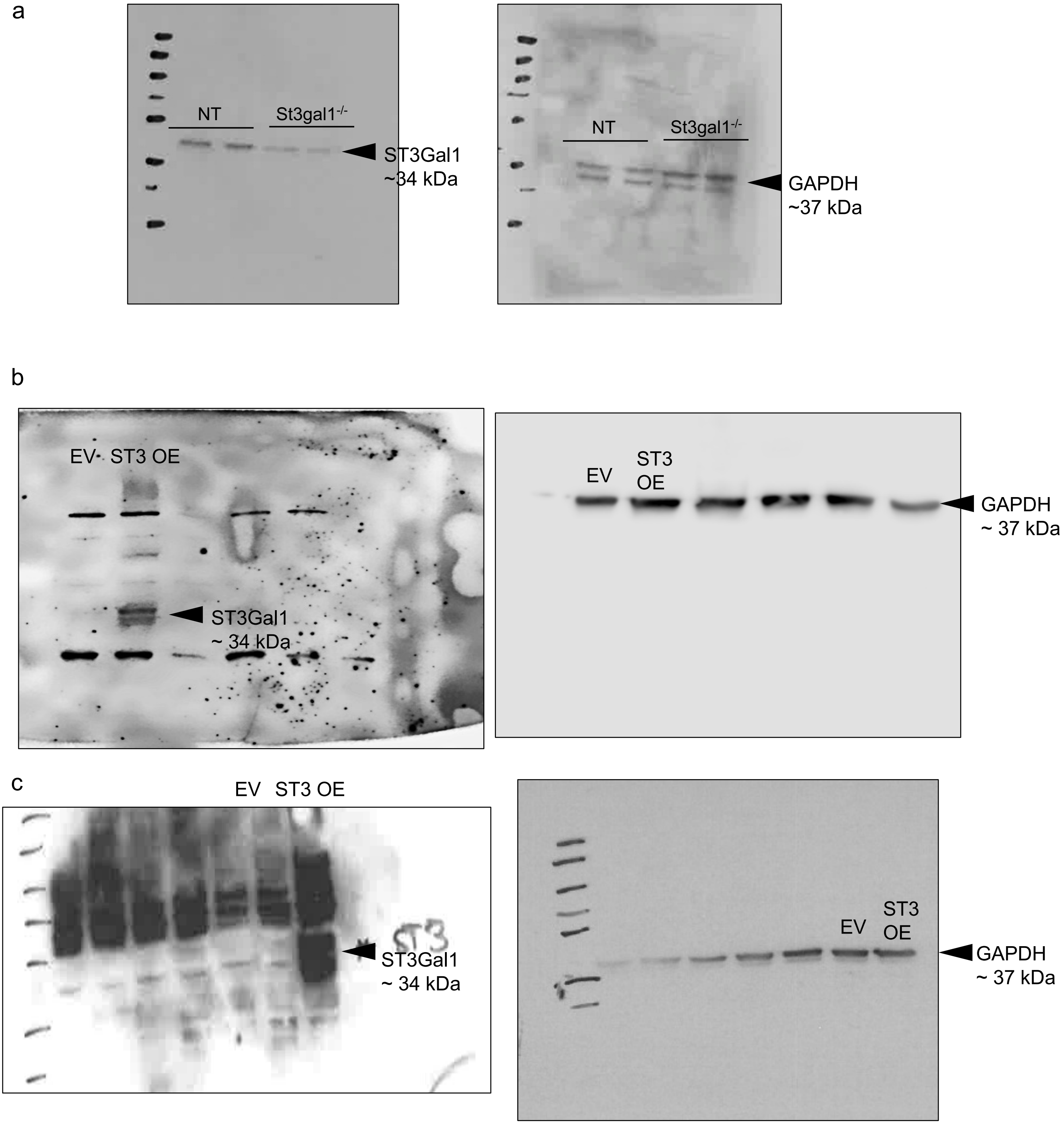

Uncropped and unedited western blots. (a) Uncropped western blot for supplementary figure 3a. (b) Uncropped western blot for supplementary figure 3h. (c) Uncropped western blot for supplementary figure 3k.

Supplementary Table 1. Table of antibodies used

Target	Supplier	Cat. Number	Dilution for IHC
AMACR	Proteintech	15918-1-AP	1 in 200
CD14	Proteintech	60253-1-Ig	1 in 200
CD163	Invitrogen	MA511458	1 in 100
GAPDH	Proteintech	60004-1-Ig	1 in 1000
Siglec-7	Proteintech	13939-1-AP	1 in 100
Siglec-9	Proteintech	13377-1-AP	1 in 200
ST3Gal1	Invitrogen	PA5-21721	1 in 500

Supplementary Table 2. Table of lectins used

Lectin	Supplier	Cat. numbers
MAL-II	Vector Laboratories	B-1265-1

Supplementary Table 3. Table of primers used

Target	Species	Forward	Reverse
ST3GAL1	human	GACACCCACACCCCTGTATTC	CTGAAAATGGTACCAACACGGC
GAPDH	human	AACAGCGACACCCATCCTC	AGCACAGCCTGGATAGCAAC
ACTIN	human	CATCGAGCACGGCATCGTCA	TAGCACAGCCTGGATAGCAAC
SIGLEC-E	mouse	TCCACAGAGCAGTGCAACTT	TGTAGGATCTTACTGCCAGGAG
GAPDH	mouse	GGCTGTATTCCCCTCCATCG	CCAGTGGTAACAATGCCATGT
ACTIN	mouse	GCACAGTCAAGGAAGAGAAT	GCCTTCTCCATGGTGGTGAA

Supplementary Table 4. Data availability of publicly available datasets used in this manuscript

Figure	Accession
Figure 1b, Figure 1i, Figure 5f, Figure 5g, Supplementary Figure 1c, Supplementary Figure 5f	TCGA PRAD (accessed via CBioPortal)
Figure 1f	GSE147250
Figure 1g	GSE35988
Figure 1h	Accessed via Oncomine. Datasets are TCGA PRAD, Armenia et al. 2018, Abida, et al. 2019 and Grasso et al. 2012.
Figure 5a	GSE133094
Figure 5d, Figure 5e, Supplementary Figure 5d	GSE21032
Supplementary Figure 1d	SU2C, 2019. Accessed via CBioPortal
Supplementary Figure 2f, Supplementary Figure 4g	GSE150368
