# Supplementary material for: ST3 beta-galactoside alpha-2,3-sialyltransferase 1 (ST3Gal1) synthesis of Siglec ligands mediates anti-tumour immunity in prostate cancer
Source: Commun Biol. 2024 Mar 6;7:276. doi: 10.1038/s42003-024-05924-0 (PMC10918101; doi:10.1038/s42003-024-05924-0)
Supplement: Supplementary file 3 — Description of Supplementary Materials [file 42003_2024_5924_MOESM3_ESM.docx]

**Description of Additional Supplementary Files**

**File name:** Supplementary Data 1

**Description:** Source data for figures 1b, 2c and 3j
